# Mapping Network Connectivity Among Symptoms of Depression and Pain in Wuhan Residents During the Late-Stage of the COVID-19 Pandemic

**DOI:** 10.3389/fpsyt.2022.814790

**Published:** 2022-03-17

**Authors:** Yuan Yang, Shu-Fang Zhang, Bing Xiang Yang, Wen Li, Sha Sha, Fu-Jun Jia, Teris Cheung, De-Xing Zhang, Chee H. Ng, Yu-Tao Xiang

**Affiliations:** ^1^Guangdong Mental Health Center, Guangdong Provincial People's Hospital, Guangdong Academy of Medical Sciences, Guangzhou, China; ^2^Research Center for Psychological and Health Sciences, China University of Geosciences, Wuhan, China; ^3^Department of Psychiatry, Wuhan Mental Health Center, Wuhan, China; ^4^School of Nursing, Wuhan University, Wuhan, China; ^5^Shanghai Key Laboratory of Forensic Medicine, Key Laboratory of Forensic Science, Ministry of Justice, Shanghai Forensic Service Platform, Academy of Forensic Science, Shanghai, China; ^6^Beijing Key Laboratory of Mental Disorders Beijing Anding Hospital, The National Clinical Research Center for Mental Disorders, The Advanced Innovation Center for Human Brain Protection, School of Mental Health, Capital Medical University, Beijing, China; ^7^School of Nursing, Hong Kong Polytechnic University, Hong Kong, Hong Kong SAR, China; ^8^The Jockey Club School of Public Health and Primary Care, The Chinese University of Hong Kong, Hong Kong, Hong Kong SAR, China; ^9^Department of Psychiatry, The Melbourne Clinic and St Vincent's Hospital, University of Melbourne, Richmond, VA, Australia; ^10^Unit of Psychiatry, Department of Public Health and Medicinal Administration, Faculty of Health Sciences, Institute of Translational Medicine, University of Macau, Macau, Macao SAR, China; ^11^Centre for Cognitive and Brain Sciences, University of Macau, Macau, Macao SAR, China; ^12^Institute of Advanced Studies in Humanities and Social Sciences, University of Macau, Macau, Macao SAR, China

**Keywords:** COVID-19, depression, network analysis, pain, Chinese

## Abstract

**Background:**

Symptoms of depression and pain often overlap, and they negatively influence the prognosis and treatment outcome of both conditions. However, the comorbidity of depression and pain has not been examined using network analysis, especially in the context of a pandemic. Thus, we mapped out the network connectivity among the symptoms of depression and pain in Wuhan residents in China during the late stage of the COVID-19 pandemic.

**Methods:**

This cross-sectional study was conducted from May 25, 2020 to June 18, 2020 in Wuhan, China. Participants' depressive and pain symptoms were assessed using the 9-item Patient Health Questionnaire (PHQ9) and a pain numeric rating scale (NRS), respectively. Network analyses were performed.

**Results:**

In total, 2,598 participants completed all assessments. PHQ4 (fatigue) in the depression community showed the highest strength value, followed by PHQ6 (worthlessness) and PHQ2 (depressed or sad mood). PHQ4 (fatigue) was also the most key bridge symptom liking depression and pain, followed by PHQ3 (sleep difficulties). There were no significant differences in network global strength (females: 4.36 vs. males: 4.29; S = 0.075, *P* = 0.427), network structure-distribution of edge weights (M = 0.12, *P* = 0.541), and individual edge weights between male and female participants.

**Conclusion:**

Depressive and pain symptoms showed strong cross-association with each other. “Fatigue” was the strongest central and bridge symptom in the network model, while “sleep difficulties” was the second strongest bridge symptom. Targeting treatment of both fatigue and sleep problems may help improve depressive and pain symptoms in those affected.

## Introduction

Depressive disorders (depression hereafter) are the third leading cause of disability globally, after low back pain and headache disorders (1). According to the World Health Organization (WHO), around 4% of the world's population suffer from depression, which is more prevalent among females than males (2). Individuals with depression often present with somatic symptoms, like tiredness, pain and sleep disturbances (3).

Pain is recognized as having both sensory dimension (i.e., intensity) and affective dimension (such as unpleasantness, anxiety, sadness, and annoyance) (4). It could have a negative effect on one's emotions and cognitive function (4). Previous studies have found that age, gender, genetic predisposition, and cognitive and/or emotional process around pain could significantly affect the perceived pain level (3, 5). For example, males, and older adults were more likely to report higher pain levels compared to their counterparts (3).

Symptoms of depression and pain often overlap, and a growing body of literature has highlighted the interaction between both conditions (3–6). Researchers have found that depression and pain share common biological pathways involving similar neurotransmitters, and this association is often referred to as “depression-pain syndrome” or “depression-pain dyad” (4, 5). Michaelides et al. reported that there is a bidirectional link between depression and pain, that is, depression could lead to increased perception of pain, and prolonged pain could worsen depression (3). The prevalence of pain in patients with depression is high, ranging from 15 to 100% (5, 7, 8), while between 13 and 85% of patients with pain report having depressive symptoms (5, 9, 10).

Depression has a negative impact on the prognosis and treatment outcome of pain, and vice versa (4, 5). Compared to non-depressed patients with pain, patients with comorbid depression and pain tend to experience more pain complaints, more severe pain symptoms, and greater impairments (11). Depressed patients also tend to report poorer response to pain treatment than the non-depressed group (12). On the other hand, the presence of pain is associated with more depressive symptoms and worse outcomes, such as lower quality of life, poorer work performance, and higher health service utilization (5). A longitudinal study found that higher pain levels, greater number of pain locations, and longer duration of pain at baseline increased the risk of depression in the following two years (13).

The COVID-19 pandemic has negatively impacted on the mental health of the population (14–16), as result of limited interpersonal contact, isolation, loneliness, fear, uncertainty, and financial stress in their daily life (17). One systematic review found that around 31.9% [95% confidence interval (CI): 27.5–36.7%] of the general population reported anxiety, and about 33.7% (95% CI: 27.5–40.6%) reported depression (18). Studies have also found that the chronic stress of living with the COVID-19 pandemic could lead to a host of physical symptoms (19), such as somatic pain, sleep difficulties, digestive problems, hormonal imbalances, and fatigue (20). One study reported that the rate of chronic pain may have increased since the COVID-19 pandemic (17). Another study found that daily routine changes during the COVID-19 pandemic were closely associated with pain intensity and emotional distress (21).

Even though depression has a negative impact on pain, and vice versa, the interaction between both conditions has not been fully investigated (5). Past studies commonly employed latent variable model to analyse the comorbidity of two psychiatric disorders, which assumed that symptoms are not directly associated with each other, and that the comorbidity is due to the interactions between unobserved latent variables (22, 23). To the best of our knowledge, no study has examined the comorbidity of depression and pain from the perspective of network analysis.

Network analysis is widely used in psychology and psychiatry research in different populations (23). In the theory of network analysis, the focus shifts from latent variables to individual symptoms, and comorbidity occurs when symptoms from different disorders are directly linked to each other (23). Additionally, network analysis can identify bridge symptoms that trigger and maintain co-occurring disorders (24). To this end, network approach may have an advantage in examining the various associations between depression and pain.

In summary, we applied network analysis to: (1) map out the network connectivity among symptoms of depression and pain in Wuhan residents in China during the late stage of the COVID-19 pandemic; and (2) identify central symptoms and bridge symptoms in the depression-pain network.

## Methods

### Study Design and Settings

This was a cross-sectional study conducted from May 25, 2020 to June 18, 2020 in Wuhan, China. Due to the COVID-19 outbreak, face-to-face assessments were not adopted. Instead, following recently published studies (25, 26), a WeChat-based QuestionnaireStar application was used. WeChat is a widely used social communication application, with more than 1.2 billion active users in China. To be eligible, participants were: (1) aged 18 years or older; (2) able to read and understand Chinese; (3) able to provide written informed consent. In this study, snowball sampling method was used. Residents in Wuhan were invited to scan the QR code through WeChat to complete the assessment, and they were also invited to distribute the QR code to their eligible families and friends. Study participants were assured of their anonymity and confidentiality. The study protocol was approved by the ethics committee of Beijing Anding Hospital, Capital Medical University, China.

### Measurements

Participants' basic demographic data, such as age, gender, living area (rural/urban) and educational level, were collected. Depression was assessed using the 9-item Patient Health Questionnaire (PHQ-9) that measures anhedonia, depressed/sad mood, sleep difficulties, fatigue, appetite changes, feeling of worthlessness, concentration difficulties, psychomotor agitation or retardation, and thoughts of suicide. Each item is scored from 0 (not at all) to 3 (almost everyday) (27). A total score of 5 or more indicates depressive symptoms (27). The Chinese version of PHQ-9 shows good psychometric properties, with Cronbach's alpha of 0.89 (28). Participant's somatic pain level was assessed using the “0–10” numeric rating scale (NRS) (i.e., how would you rate your pain at its worst over the past 3 days?) (29), in which 0 means ‘no pain' and 10 means ‘the worst pain'. Participants were asked to select a number between 0 and 10, to indicate their current pain level. A total score of 4 or more indicates that the patient is currently suffering from pain (29).

### Statistical Analyses

#### Network Structure

Network analysis is an alternative approach to examine the comorbidity of two disorders or syndromes (30). Each symptom in the model is a “node,” whereas the link between two nodes is shown as an “edge.” In network analysis, nodes that are stronger and/or more connected with others are located in the central area of the model, while nodes with less connections are placed in the periphery of the model (31). A thicker edge indicates a higher correlation (32). To identify central symptoms in the network, three major centrality indices are usually computed: “*Strength*” (i.e., the total sum of the absolute weights of the edge connecting the node to all the other nodes), “*Betweenness*” (i.e., the number of shortest paths connecting any two symptoms), and “*Closeness*” (i.e., the inverse of the sum of the total length of all the shortest paths) (33). Additionally, to explore the role of a symptom/node as a bridge in connecting two or more psychiatric disorders or syndromes (i.e., between depression and pain), three bridge indices are usually computed: “*Bridge Strength*,” “*Bridge Betweenness*,” and “*Bridge Closeness*” (34). For each index, high values reflect great centrality or bridge centrality (35).

To explore the network structure of the association between depression and pain, the Extended Bayesian Information Criterion (EBIC) combined with the graphical least absolute shrinkage and selection operator (LASSO) method was used (36). All the analyses were conducted using R program (Version 4.0.3) (37). “*Bootnet”* (38) and “*qgraph”* (32) packages were utilized to generate the network and test network stability and accuracy. The predictability (defined as the variance in a node that is explained by all other nodes in the network) of each node was estimated using the “*mgm*” package (39). Following previous studies (40, 41), the “*estimateNetwork*” function was adopted to assess the network model, with “*EBICglasso*” as the default method, and 0.5 as the default tuning parameter (38). The green color of edge indicates a positive correlation, while the red color indicates a negative correlation (32).

#### Central Symptoms and Bridging Symptoms

Centrality indicator (*Strength*) was calculated using the “*centralityPlot*” function in the “*qgraph*” package (38). The bridge centrality (*Bridge Strength*) of each node was explored by the “*bridge*” function in “*networktools*” package (23, 35). Recent studies have recommended that both *Betweenness* and *Closeness* centrality might not be robust and trustworthy in psychological networks (42). Thus, in the subsequent network analysis, we focused on *strength* (43). Similarly, in terms of bridge centrality, *bridge strength* is considered as the most appropriate index to identify bridge symptoms (34, 44).

#### Network Stability and Accuracy

To examine the stability and accuracy of the networks (38), a case-dropping bootstrap procedure was performed to compute correlation stability coefficient (CS-C) (1,000 replicates, 8 cores). A CS-C (correlation = 0.7) represents the maximum percentage of sample cases that can be dropped from the original full study sample to retain a correlation of 0.7 between the original centrality indices and the centrality indices based on case-subset network in at least 95% of the samples (38). The CS-C is required to be above 0.25, and preferably 0.50 (38). Next, non-parametric bootstrapping method (1,000 replicates, 8 cores) was used to estimate the accuracy of edge-weights by computing confidence intervals (CIs). Larger CIs indicate poorer precision in the estimation of edges, while narrower CIs indicate a more trustworthy network (43). Finally, differences in the network's properties (i.e., edge weight, and node strength) were evaluated by bootstrapped difference tests (38).

#### Network Comparison Between Genders

Previous evidence indicated that gender is significantly associated with depression and pain (3). Therefore, following previous network analyses (44, 45), the differences of network characteristics between male and female participants were compared, using the R “*NetworkComparisonTest”* package (Version 2.2.1) (46). This test was conducted in subsamples (i.e., females vs. males) with 1,000 permutations. Possible difference at the level of network structure (distributions of edge weights), global strength (total absolute connectivity among the symptoms), and of each specific edge between both gender groups were examined.

## Results

### Study Sample

Of the 2,614 individuals invited to participate in this study, 2,598 (99.4%) participants fulfilled the study entry criteria and completed all the assessments. Of them, 1,930 (74.3%) were females and 668 (25.7%) were males. The mean age of the sample was 35.5 [standard deviation (SD) = 10.9 years], ranged from 18 to 88 years. The majority of the participants had high educational background (college and above, *n* = 2,367, 91.1%), and lived in urban areas (*n* = 2,280, 87.8%). A total of 1,231 (47.4%) participants reported depressive symptoms, and 1,292 (49.7%) of participants were currently suffering from pain.

### Network Structure

**Figure 1:**
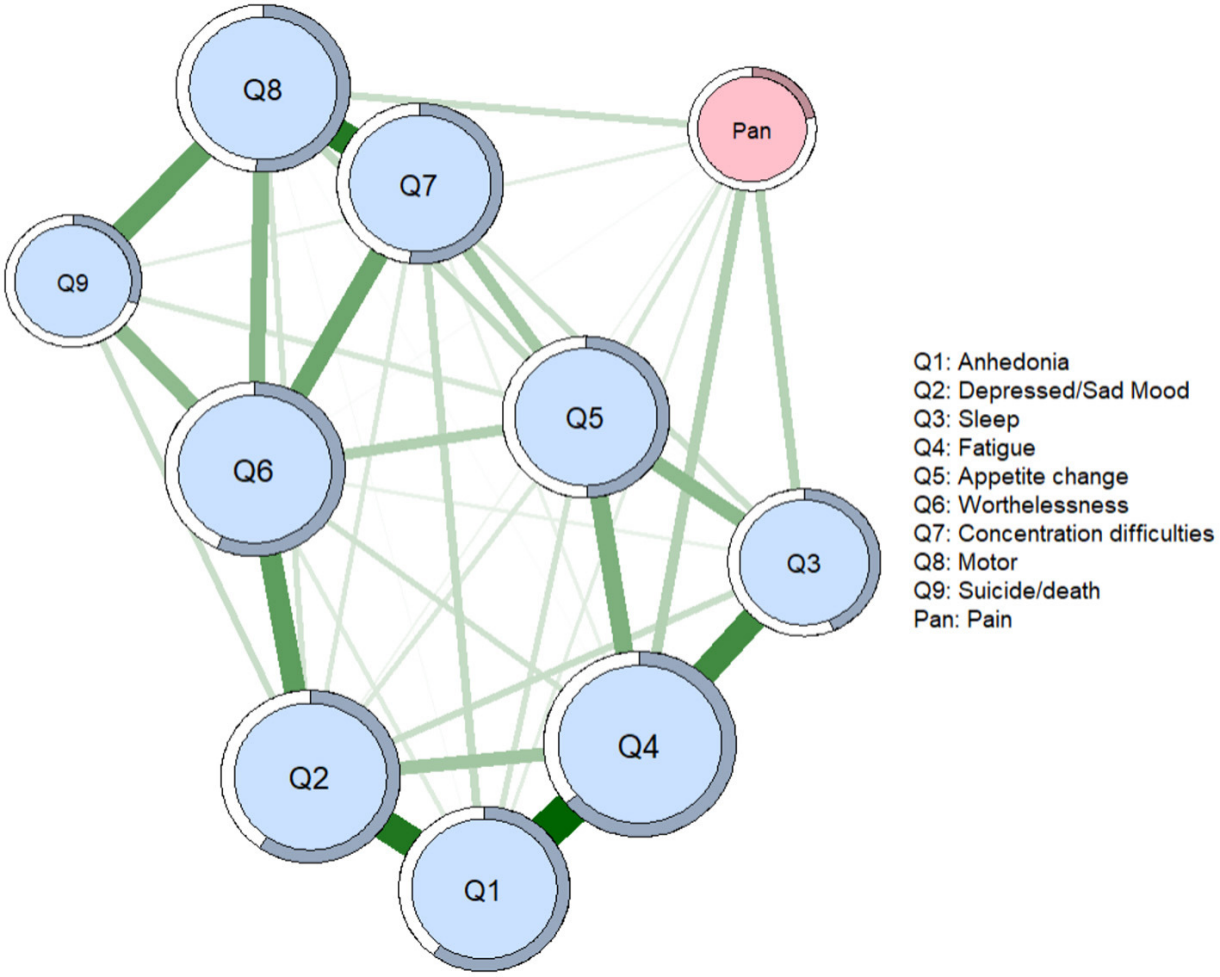
Network of symptoms of depression and pain. In the diagram, pink node represents pain, and light blue nodes represent 9 depressive symptoms. Nodes with stronger correlations are closer to each other. The thickness of an edge indicates the strength of the correlation. Q = question from the Patient Health Questionnaire; Green lines = positive associations.

Figure 1 presents the network structure of depression and pain symptoms among Wuhan residents during the late stage of the COVID-19 pandemic. Forty out of the 45 (88.89%) edges were above zero. The model showed that the edges between PHQ1 (anhedonia) and PHQ4 (fatigue), between PHQ1 (anhedonia) and PHQ2 (depressed/sad mood), and between PHQ7 (concertation difficulties) and PHQ8 (psychomotor change) were the three strongest positive edges in the depression community. Additionally, the edges between PHQ4 (fatigue) and pain was the strongest positive edge liking the depression community and self-perceived pain, followed by the edge between PHQ3 (sleep difficulties) and pain, and PHQ8 (psychomotor change) and pain. The correlation matrices are showed in Supplementary Table 1.

### Central Symptoms and Bridging Symptoms

**Figure 2:**
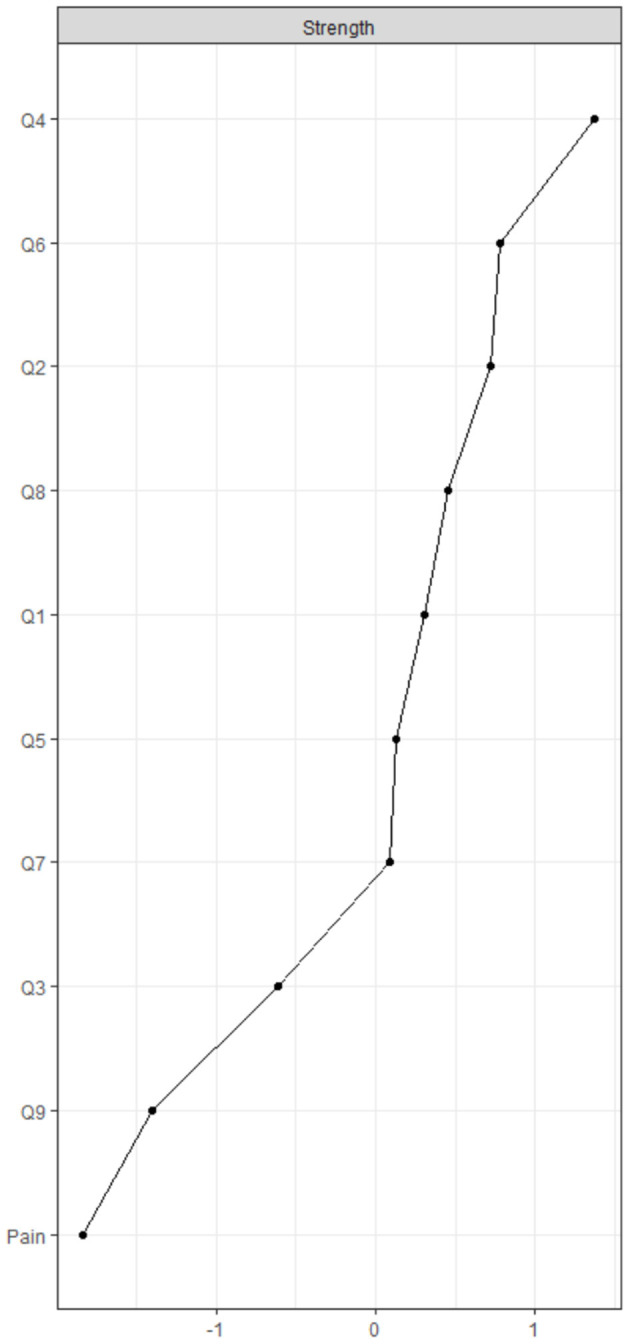
Centrality indices of network. Q = question from the Patient Health Questionnaire.

**Figure 3:**
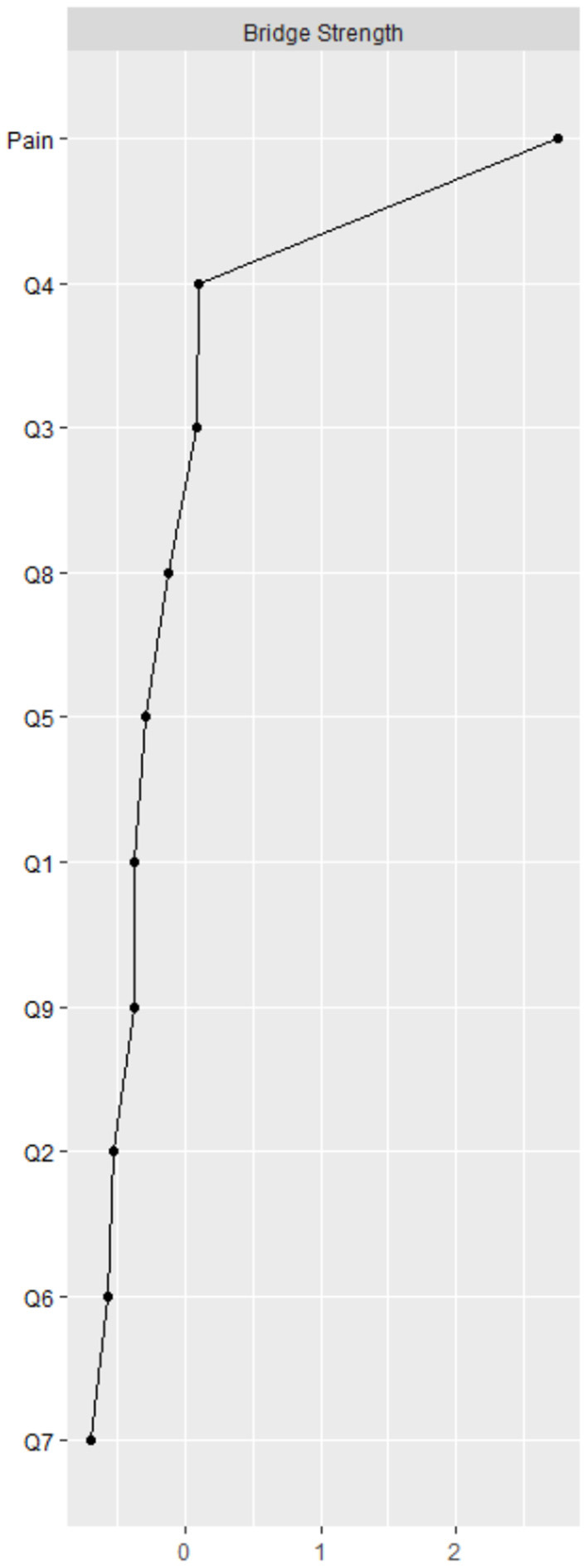
Bridge centrality indices of network. Q = question from the Patient Health Questionnaire.

According to the centrality indices, PHQ4 (fatigue, strength index = 1.162) has the highest strength value, followed by PHQ6 (worthlessness, strength index = 1.032) and PHQ2 (depressed or sad mood, strength index = 1.019). Additionally, in the depression community, PHQ9 (suicide/death, strength index = 0.557) has the lowest strength value (Figure 2 and Table 1). In terms of bridge centrality, PHQ4 (fatigue, bridge strength index = 0.106) in the depression community was the most key bridge symptom liking depression and pain, followed by PHQ3 (sleep difficulties, bridge strength index = 0.104). In contrast, PHQ7 (concertation difficulties, bridge strength index = 0) has the lowest bridge strength value in the depression community (Figure 3 and Table 1).

**Table 1:** Centrality estimates of nodes in the network.

	**Strength**	**Bridge strength**	**Predictability**
Q1 (Anhedonia)	0.928	0.042	0.601
Q2 (Depressed/sad mood)	1.019	0.022	0.597
Q3 (Sleep)	0.729	0.104	0.436
Q4 (Fatigue)	1.162	0.106	0.639
Q5 (Appetite change)	0.890	0.054	0.498
Q6 (Worthlessness)	1.032	0.015	0.569
Q7 (Concentration difficulties)	0.881	0	0.520
Q8 (Motor)	0.960	0.077	0.513
Q9 (Suicide/death)	0.557	0.042	0.306
Pain	0.462	0.462	0.219

*Predictability: proportion of the variance explained by all other symptoms in the network, Q = question from the Patient Health Questionnaire*.

### Network Stability and Accuracy

**Figure 4:**
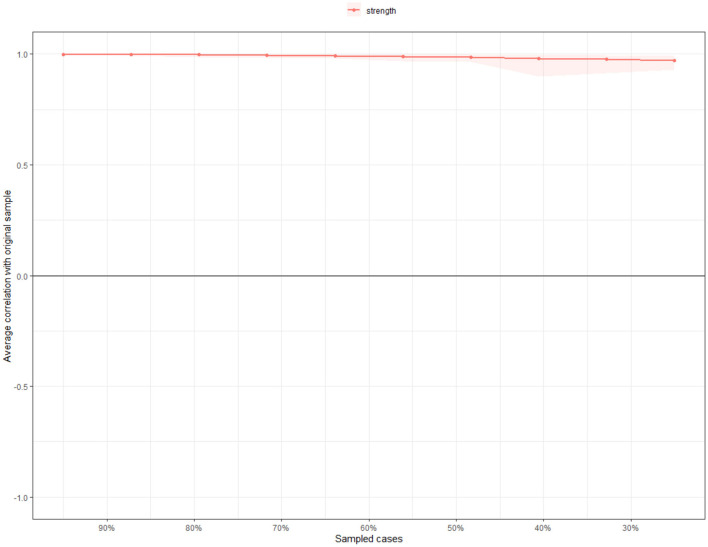
Stability of network structure by case dropping subset bootstrap. The X-axis represents the percentage of cases of original sample used at each step. The Y-axis represents the average of correlations between the centrality indices from the original network and the centrality indices from the networks that were re-estimated after dropping increasing percentages of cases. Colored areas indicate 95% confidence interval.

The case dropping bootstrap procedure shows that the value of strength, betweenness, and closeness remained stable after dropping different proportions of the sample (Figure 4). The CS-Cs for strength, betweenness, and closeness were 0.75, 0.28, and 0.75, respectively, all exceeding the recommended threshold of 0.25 (38). The CS-C for strength was 0.75, indicating that even after dropping 75% of the sample, the results did not change significantly compared to the original results.

The results of the bootstrap 95% CI for edges and bootstrapped differences tests for edge weight and node strength are shown in the Supplementary Material. The bootstrapped 95% CIs for estimated edge weights were relatively narrow, indicating that most edges were stable and accurate (Supplementary Figure 1). Significance tests of edge weight differences indicated that the three thickest and most saturated edges were significantly stronger than most of the other edges in the network (43): PHQ1 (anhedonia) - PHQ4 (fatigue), PHQ1 (anhedonia) - PHQ2 (depressed or sad mood), and PHQ7 (concertation difficulties) - PHQ8 (psychomotor change) (Supplementary Figure 2). Significance tests of node strength differences indicated that PHQ4 (fatigue) was significantly stronger than other nodes in the network, followed by PHQ6 (worthlessness) and PHQ2 (depressed or sad mood) (Supplementary Figure 3).

### Network Comparison Between Genders

The network characteristics were compared between males and females. We found that between both genders, there were no significant differences in network global strength (females: 4.36 vs. males: 4.29; S = 0.075, *P* = 0.427), network structure-distribution of edge weights (M = 0.12, *P* = 0.541), and individual edge weights (*P* all > 0.05 after Holm-Bonferroni correction) (Supplementary Figure 4).

## Discussion

This study mapped out the network connectivity among symptoms of depression and pain in Wuhan residents during the late stage of the COVID-19 pandemic, and identified the central and bridge symptoms in the network. The network model confirmed results of previous studies that depression is strongly associated with the perception of pain (3). Several edges found in our study have also been reported in past studies. For example, our study found a strong link between depression items PHQ1 (anhedonia) and PHQ4 (fatigue), PHQ1 (anhedonia) and PHQ2 (depressed or sad mood), PHQ3 (sleep difficulties) and PHQ4 (fatigue), and PHQ7 (concentration difficulties) and PHQ8 (psychomotor agitation or retardation), which is consistent with past network analysis findings among different populations (22, 41, 47, 48).

### Central Symptoms

In the current study, we found that PHQ4 (fatigue) has the highest strength value. Significance tests of node strength differences also indicated that “fatigue” was significantly stronger than other nodes in the network. This is similar to past studies, which found that PHQ4 “fatigue” had the highest strength among depression and anxiety symptoms in different populations (41, 47). Additionally, it has been reported that in Asian populations, people tended to express their distress as somatic symptoms (e.g., fatigue, and insomnia) instead of emotional symptoms (49). A systematic review concluded that the “typical” presentation of depression in primary care is dominated by somatic complaints, with over 50% of patients with depression reporting somatic complaints only, including fatigue (5). Besides, the centrality of “fatigue” may be attributed to the COVID-19 pandemic. As Wuhan was the epicenter of the disease in China with high infection and mortality rates, residents in Wuhan were more likely to experience fear, uncertainty, and anxiety compared to people in other provinces in China (50). Majumdar et al. reported that the COVID-19 pandemic with mass lockdown was a key cause of high levels of stress, insomnia, pain, and fatigue among the general population (20), which partially supports our findings.

In the current study, PHQ6 (worthlessness), and PHQ2 (depressed or sad mood) were the second and third strongest nodes in the network. The findings are consistent with the conventional concept where “sad mood” is the hallmark symptom of depression (40, 51–53). Also, as depression progresses, helplessness, hopelessness, and worthlessness may occur, which is known as the cognitive triad of depression (54). According to the Diagnostic and Statistical Manual of Mental Disorders (DSM−5), “depressed mood” (e.g., feels sad, empty, hopeless, tearful) and “loss of interest or pleasure” are core symptoms for the diagnosis of depression (55), and “depressed mood” is also the most important negative mood to maintain depression (56). On the contrary, the least central symptom found in our study was PHQ9 (suicide/death). Previous network studies found conflicting findings regarding the centrality of suicide/death; some reported it has high strength index (57), while others found it has low centrality (52, 53, 58).

In summary, central symptoms such as “fatigue,” “worthlessness,” and “depressed or sad mood” are critical and may be potential targets for further interventions, since these symptoms are most likely to influence the whole model of comorbid depression and pain. Previous studies found that patients who endorsed more central symptoms of depression at baseline were more likely to experience major depressive disorder in the future, compared to those who endorsed more peripheral symptoms (59). Therefore, targeting the central symptoms may improve treatment effectiveness.

### Bridge Symptoms

There were two important bridge symptoms between depression and pain, namely, “fatigue” and “sleep difficulties.” This study indicates that “fatigue” is not only the strongest and most central symptom in the network but is also the most important node that acts as a link between depression and pain communities. Our findings are partially concordant with previous network analyses which found that “fatigue” and “sleep difficulties” were important bridge symptoms between depression and anxiety disorders (41, 47).

“Fatigue,” “sleep difficulties” and “pain” all fall under the physiological dimension rather than the affective dimension of a disorder, and this may explain the strong intertwined links among them (6). Patients with chronic fatigue syndrome are more likely to report insomnia, muscle and/or joint pain, and headaches (60). Further, insomnia is the major source of distress in individuals with chronic pain, and it could also increase fatigue (61). The relationship between sleep and pain is likely to be bidirectional (61). Untreated insomnia can serve to increase pain perception and pain can worsen sleep (61, 62). Moreover, the physical and emotional efforts to cope with pain can aggravate fatigue and lead to poor sleep quality (6). In other words, fatigue, insomnia, and pain are all strongly intertwined with each other. Further, the activation of bridge symptoms is likely to trigger and maintain the comorbidity of two disorders or syndromes (24). Thus, our findings suggest that “fatigue” and “sleep problems,” being the key cross-association symptoms, may be potential targets in future treatment strategies.

### Network Stability and Network Comparison

In our study, both network edges and the centrality metric were stable, which increased the reliability in drawing conclusion from this cross-sectional network (40). Network comparison analysis revealed that there were no significant differences in network global strength and edge weight between males and females, indicating that gender did not significantly influence the association between depression and pain. Previous evidence have indicated that females were more likely to report depression than males (2), while males were more likely to report higher pain levels than females (3). It is possible that the two-opposing influencing forces of gender offset each other resulting in non-significant difference.

### Strengths and Limitations

To the best of our knowledge, this was the first network analysis among symptoms of depression and pain in Wuhan residents during the late stage of the COVID-19 pandemic. The strength of this study included a large sample size, and the use of standardized assessment instruments and sophisticated network approach. However, several limitations should be noted. First, due to the cross-sectional study design, the causal relationship or the dynamic changes between depression and pain could not be examined. Second, certain biological factors which may influence the link between depression and pain, such as neurotransmitter levels, were not examined in this study. Third, the results should be interpreted with caution as the generated networks were based on group-level analysis, and whether group-level results can represent individuals remained unclear (63).

## Conclusion

In conclusion, depression and pain symptoms showed strong cross-association with each other in this study. “Fatigue” was the strongest central and bridge symptom in the network model. “Worthlessness” and “depressed or sad mood” were the second and third strongest central node in the network, while “sleep difficulties” was the second strongest bridge symptom in the model. Gender did not significantly influence the association between depression and pain. Hence, these central and bridge symptoms are potential targets to improve treatment effectiveness for those with comorbid depression and pain. In addition, individuals with such symptoms should be screened and monitored carefully to prevent worsening of comorbid depression and pain. Furthermore, longitudinal studies are recommended in the future to confirm the findings of this study.

## Data Availability Statement

The Clinical Research Ethics Committee of participating hospitals that approved the study prohibits the authors from making the research dataset of clinical studies publicly available. Readers and all interested researchers may contact Dr. Y-T Xiang (Email address: xyutly@gmail.com) for details. Dr. Xiang will apply to the Clinical Research Ethics Committee of participating hospitals for the release of the data.

## Ethics Statement

The studies involving human participants were reviewed and approved by Beijing Anding Hospital, Capital Medical University, China. The patients/participants provided their written informed consent to participate in this study.

## Author Contributions

S-FZ, BY, and Y-TX: study design. YY, S-FZ, BY, WL, SS, F-JJ, TC, and D-XZ: data collection, analysis, and interpretation. YY and Y-TX: drafting of the manuscript. CN: critical revision of the manuscript. All authors approved of the final version for publication.

## Funding

The study was supported by the National Science and Technology Major Project for investigational new drug (No. 2018ZX09201-014), the Beijing Municipal Science and Technology Commission (No. Z181100001518005), the National Social Science Foundation of China (No. 19ZDA360), the University of Macau (No. MYRG2019-00066-FHS), and the Fundamental Research Funds for the Central Universities (No. 2020YJ065).

## Conflict of Interest

The authors declare that the research was conducted in the absence of any commercial or financial relationships that could be construed as a potential conflict of interest.

## Publisher's Note

All claims expressed in this article are solely those of the authors and do not necessarily represent those of their affiliated organizations, or those of the publisher, the editors and the reviewers. Any product that may be evaluated in this article, or claim that may be made by its manufacturer, is not guaranteed or endorsed by the publisher.
