# Supplementary material for: Mapping Network Connectivity Among Symptoms of Depression and Pain in Wuhan Residents During the Late-Stage of the COVID-19 Pandemic
Source: Front Psychiatry. 2022 Mar 17;13:814790. doi: 10.3389/fpsyt.2022.814790 (PMC8968182; doi:10.3389/fpsyt.2022.814790)
Supplement: Supplementary file 1 [file Data_Sheet_1.pdf]

Supplementary Figure 1. Bootstrapped confidence intervals of edge weights.

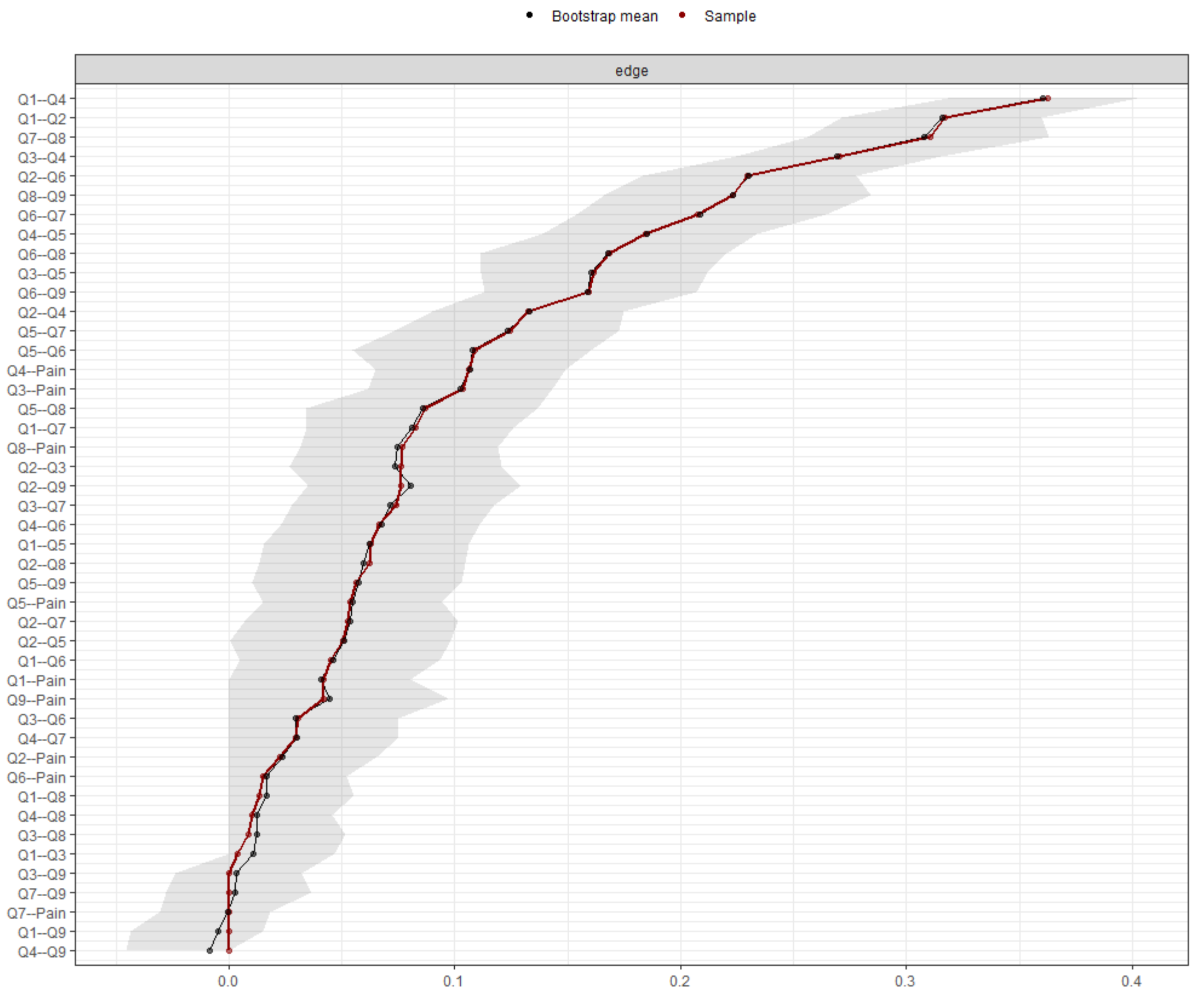

Note: The black dots indicate the values of each edge weight, ordered from the highest to the lowest value. The gray area represents the 95% confidence intervals of edge weights, estimated with the non-parametric bootstrap procedure. Wide intervals indicate lower stability and narrow intervals indicate higher stability. Q=question from the Patient Health Questionnaire.

Supplementary Figure 2. Edge weight difference by bootstrapped difference test

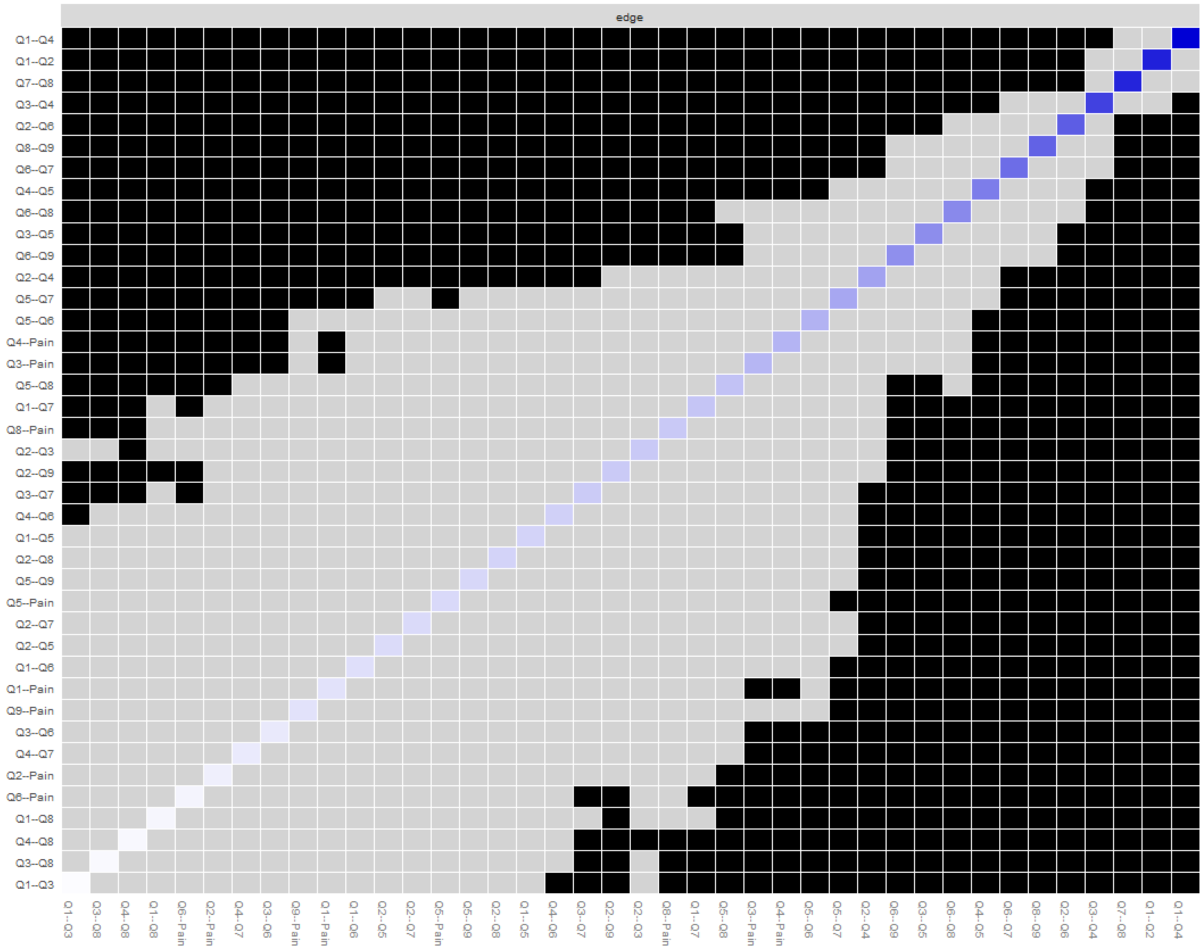

Note: Bootstrapped difference tests between edge weights in the network. Gray boxes indicate edges that do not significantly differ from one-another. Black boxes represent edges with significant difference from one another. Blue boxes in the edge-weight plot indicate positive correlations. Q=question from the Patient Health Questionnaire.

Supplementary Figure 3. Node strength difference by bootstrapped difference test.

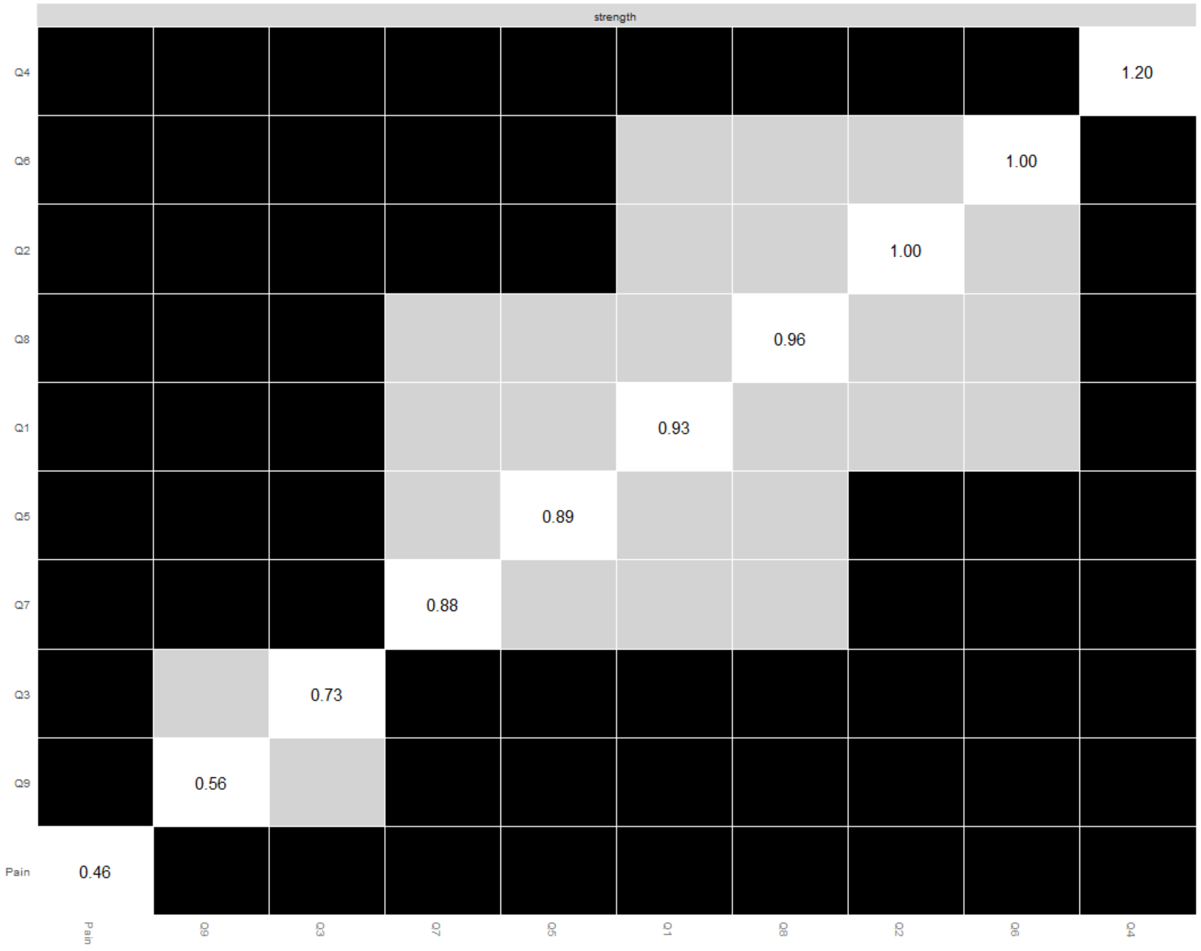

Note: Bootstrapped difference tests between node strength of factors. Gray boxes indicate nodes that do not significantly differ from one-another. Black boxes represent nodes that differ significantly from one another. White boxes show the values of node strength. Q=question from the Patient Health Questionnaire.

Supplementary Figure 4. Estimated network of symptoms of depression and pain in males and females.

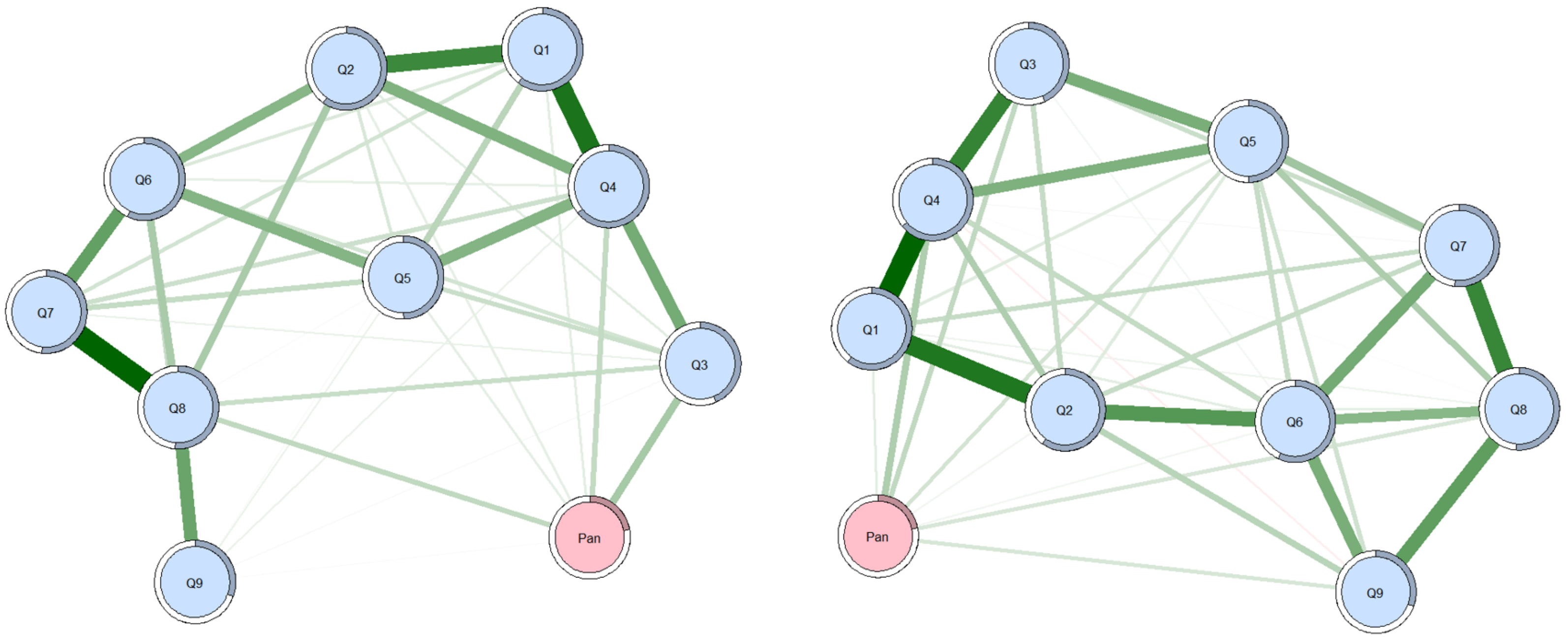

Note: left plot: female network (n=1,930); right plot: male network (n=668). In the diagram, pink node represents pain, and light blue nodes represent 9 depressive symptoms. Nodes with stronger correlations are closer to each other. The thickness of an edge indicates the strength of the correlation. Pan=pain; Q=question from the Patient Health Questionnaire; Green lines=positive associations.

Supplementary Table 1. Edge weight matrix

	Q1	Q2	Q3	Q4	Q5	Q6	Q7	Q8	Q9	Pain
**Q1 (Anhedonia)**	0									
**Q2 (Depressed/Sad Mood)**	0.317	0								
**Q3 (Sleep)**	0.004	0.076	0							
**Q4 (Fatigue)**	0.362	0.132	0.270	0						
**Q5 (Appetite change)**	0.063	0.050	0.162	0.185	0					
**Q6 (Worthlessness)**	0.045	0.230	0.031	0.067	0.109	0				
**Q7 (Concentration difficulties)**	0.082	0.053	0.074	0.029	0.124	0.208	0			
**Q8 (Motor)**	0.013	0.062	0.009	0.010	0.087	0.168	0.310	0		
**Q9 (Suicide/death)**	0	0.076	0	0	0.057	0.159	0	0.223	0	
**Pain**	0.042	0.022	0.104	0.106	0.054	0.015	0	0.077	0.042	0
Note: Q=question from the Patient Health Questionnaire										
